# Isolation of *Acanthamoeba* Species and Bacterial Symbiont Variability in Puna Salt Plains, Argentina

**DOI:** 10.1111/1758-2229.70059

**Published:** 2025-01-14

**Authors:** Ronnie Mooney, Kiri Rodgers, Sandro Carnicelli, Matías E. Carnevale, Maria Eugenia Farias, Fiona L. Henriquez

**Affiliations:** ^1^ Department of Civil and Environmental Engineering University of Strathclyde Scotland UK; ^2^ School of Health and Life Sciences University of the West of Scotland Lanarkshire Scotland UK; ^3^ School of Business and Creative Industries University of the West of Scotland Lanarkshire Scotland UK; ^4^ Centro Regional de Energía y Ambiente Para el Desarrollo Sustentable (CREAS) CONICET/UNCA Tucumán Argentina; ^5^ PunaBio S.A., Campus USP‐T Tucumán Argentina; ^6^ PunaBio and CONICET Andean Lagoons Microbiological Research Laboratory (LIMLA‐PROIMI) San Miguel de Tucumán Argentina

**Keywords:** *Acanthamoeba*, intracellular, microbiome, symbiosis

## Abstract

*Acanthamoeba* spp. are widespread protists that feed on bacteria via phagocytosis. This predation pressure has led many bacteria to evolve strategies to resist and survive inside these protists. The impact of this is not well understood, but it may limit detection and allow survival in extreme environments. Three sites in the Puna salt plains, Catamarca province, Argentina, were sampled for *Acanthamoeba* spp., verified using PCR and Sanger sequencing. The intracellular microbiome was analysed with 16S rRNA gene sequencing and compared to the overall site microbiome. *Acanthamoeba* were found at all locations, and their intracellular microbiome was similar across samples but differed from the overall site microbiome. *Pseudomonas* spp., a clinically relevant genus, was most abundant in all isolates. This study suggests *Acanthamoeba* can protect bacteria, aiding their detection avoidance and survival in harsh conditions.

## Graphical abstract

*Acanthamoeba* isolated from the extreme environments of the Puna salt plains in Argentina contain unique intracellular microbial communities that are distinct from those found in the surrounding environments. The number of shared taxa amongst intracellular amoebae across all sites was greater than the number of shared taxa between the amoebae and their respective environments.

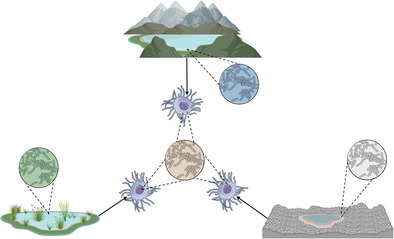

## 1 Introduction

Microbial co‐existence within environmental communities is becoming increasingly relevant both clinically and economically. Whilst the consequences of interactions between bacteria are well documented, such as the transfer of antimicrobial resistance genes (Baker et al. 2018; Ma, Konkel, and Lu 2021; McCarthy et al. 2014; Michaelis and Grohmann 2023; Zhu, Huang, and Yang 2022), the role of predatory protists within the environment is often overlooked. The active predation of bacteria in an environment by protists is essential in maintaining soil health and diversity (Clarholm 1981). However, the selective pressures incurred have given rise to a variety of complex interactions that have significant impacts on human health (Dobrowsky, Khan, and Khan 2017; García et al. 2007; Henriquez et al. 2021; José Maschio, Corção, and Rott 2015; Leong et al. 2022; Okubo et al. 2018). The free‐living amoebae (FLA) are an ecologically diverse group of predatory protists that feed on bacteria and other microorganisms via phagocytosis (Alsam et al. 2005; Chambers and Thompson 1976), yet several bacteria have evolved sophisticated strategies for evading and resisting the phagocytic processes and can instead survive intracellularly (Flieger et al. 2018; Henriquez et al. 2021; Rayamajhee et al. 2021; Rayamajhee et al. 2022; Rayamajhee, Willcox, Sharma et al. 2024). Understanding the implications of co‐existence between amoebae and bacteria is important in furthering our understanding of pathogenesis, antimicrobial resistance, and in ensuring current detection and disinfection strategies are sufficient in eliminating the hidden microbiome within FLA.

Of particular interest are the FLA within the genus *Acanthamoeba*, given their ubiquitous distribution and propensity to harbour multiple different bacterial species (Rayamajhee et al. 2021). *Acanthamoeba* exist in both natural and human‐made environments and in themselves are opportunistic pathogens causing several infections, most notably the corneal infection *Acanthamoeba* keratitis (AK) (Culbertson, Smith, and Minner 1958; Lorenzo‐Morales, Khan, and Walochnik 2015; Somani, Ronquillo, and Moshirfar 2024). The wide distribution of *Acanthamoeba* is owed in part to the protists' ability to convert to a highly resistant cyst stage. This cyst stage can allow prolonged survival in harsh environmental conditions as well as limiting the efficacy of many commonly used disinfection strategies (Lloyd et al. 2001; Lorenzo‐Morales, Khan, and Walochnik 2015; Mooney et al. 2024; Sriram et al. 2008). The reduced antimicrobial efficacy against *Acanthamoeba* is problematic in that it can permit the survival of other pathogenic species such as *Legionella* or *Pseudomonas* (Dey et al. 2019; Dobrowsky, Khan, and Khan 2017; García et al. 2007; José Maschio, Corção, and Rott 2015; Leong et al. 2022; Mungroo, Siddiqui, and Khan 2021; Rayamajhee, Willcox, Henriquez et al. 2024; Sarink et al. 2020) whilst also limiting the detectability of these organisms using culture based and molecular approaches (Henriquez et al. 2021; Mooney et al. 2024). Indeed, bacteria within the amoebae or ‘endosymbiotic bacteria’ can tolerate higher levels of exposure to commonly used treatments such as heat or chlorine (Dobrowsky, Khan, and Khan 2017; Sarink et al. 2020), thus allowing survival in high‐risk areas (Thomas et al. 2010).

Whilst it is widely considered that FLA have the potential to permit survival of a unique intracellular microbiome in instances of extreme environmental stress, it has yet to be demonstrated in a real‐world scenario. High‐altitude salt brines, such as those found in the salt plains and lagoons of the Catamarca province, Argentina, serve as reservoirs for extreme microbial ecosystems, including microbial mats and microbialites (Boidi et al. 2020). These environments, influenced by volcanic activity, present conditions that resemble the early Earth and potentially extraterrestrial conditions (Vignale et al. 2022) and harbour a diverse range of microbial communities. As such, we have sampled three sites from the salt plains and lagoons of the Catamarca province, Argentina, considered to be an extreme environment, due to high altitude, salinity, high UV radiation, temperature fluctuations and geochemistry (Farías 2020; McGenity and Oren 2012; Vignale et al. 2022). The study of these poly‐extremophiles provides valuable insights into the molecular mechanisms underlying their resistance ability against UV and toxic or deleterious chemicals that could be useful in agricultural practices. The importance of the unique microbiomes within these ecosystems has been highlighted, however little is known about predatory protist species such as FLA within these systems or the bacteria surviving intracellularly.

In the present study, we aimed to further understand the potential of predatory protists to harbour microbiomes distinct from those of the surrounding environment, particularly in harsh environments, using a combined culture based and molecular approach. Using three uniquely extreme environments we demonstrate that *Acanthamoeba* spp. are present in the microbial community and can remain viable despite being subject to significant geochemical and environmental extremes. We also show that in doing so, they can act as a reservoir for bacteria capable of evading phagocytosis, the majority of which are not detected in the surrounding environment. Understanding the interactions of these organisms at an environmental level provides a platform for identifying risk factors prior to their emergence in clinical settings.

## 2 Methods

### 2.1 Sample Sites

**FIGURE 1:**
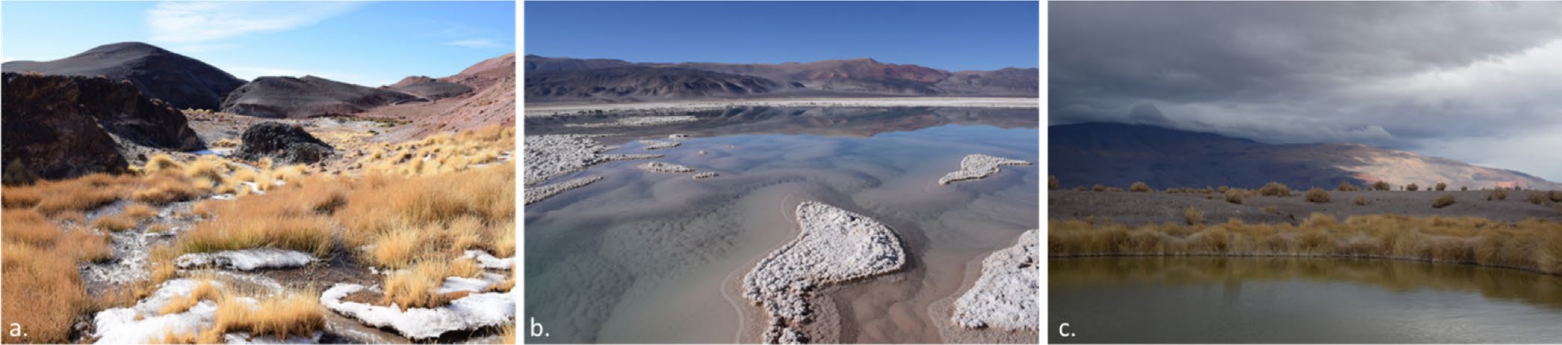
(a) Vega Colorada; (b) Laguna Verde; (c) Ojos de Campo.

Soil and water samples were collected in July 2019 across three sites of the salt flat plains and lagoons in the Andean region of the Catamarca province, in northwestern Argentina. This province is generally mountainous with intermontane tablelands and valleys (some fertile, others completely arid). Site 1 – Vega Colorada (VC; 25°35′39.69″ S, 67°30′49.44″ W) is marshy wetland, dominated by grass‐like vegetation and icy water (Figure 1a). Site 2 – Laguna Verde (LV; 25°29′08.4″ S, 67°32′13.3″ W) is a high altitude and highly saline lake, surrounded by active volcanoes and fed by hot‐springs (Figure 1b). Site 3 – Ojos de Campo (OC; 25°33′52.47″ S, 67°38′30.08″ W) consists of lagoons located within the Antofalla salt flat, an isolated desert known for lithium mining (Figure 1c). Samples were collected from the surface to a depth of 3 cm and stored in 50 mL polyethylene sterile tubes and maintained at 4°C prior to undergoing amoeba isolation and bacterial DNA extractions.

### 2.2 Isolation of *Acanthamoeba*

**FIGURE 2:**
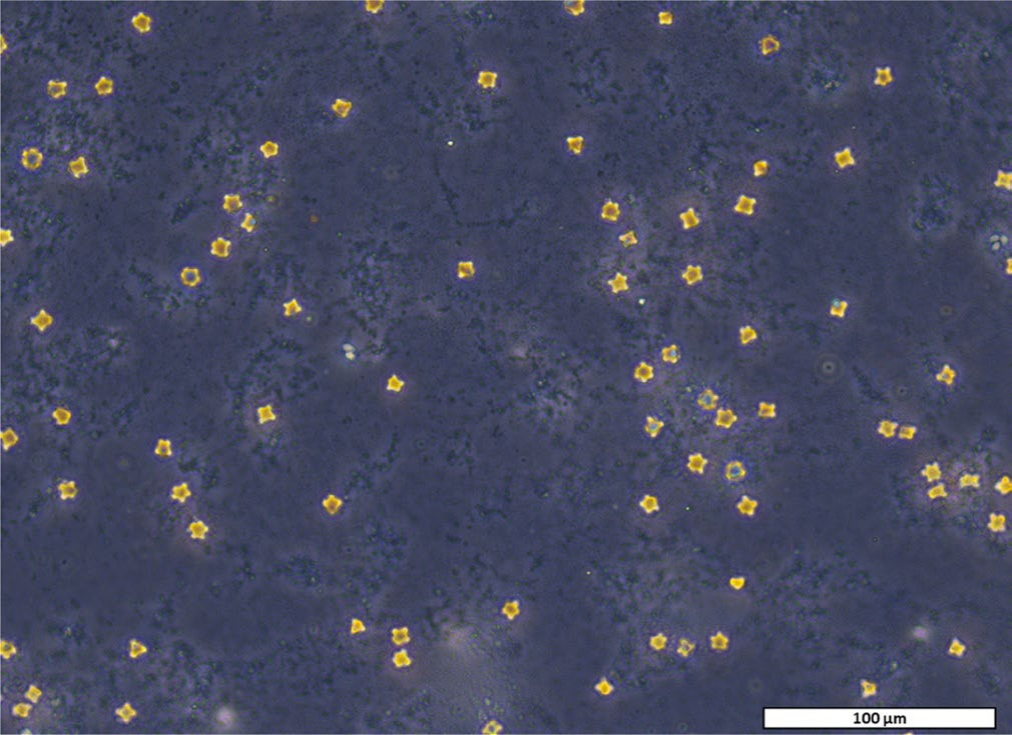
*Acanthamoeba* cysts isolated from Laguna Verde in phase contrast. Scale bar: 100 μm.

The isolation of *Acanthamoeba* species from sediment was achieved by partially streaking 50–100 mg on non‐nutrient agar plates containing PAGEs saline solution (Page 1978), covering approximately 25% of the plate surface, and allowing a minimum of 24 h for amoebae to migrate away from the streaked sediment. Plates were checked daily using an inverted microscope for the presence of cysts that were morphologically similar to *Acanthamoeba* spp. based on endocyst and ectocyst shape (Page 1978, 1988) and had migrated sufficiently from the initial streaking (Figure 2). At this point, cells were transferred to a new non‐nutrient agar plate and the process repeated one more time. Successfully isolated cells were added to a 24‐well plate with a modified media (minimal amoebic detection [MAD] media; potassium dihydrogen orthophosphate – 360 mg/L, methionine – 300 mg/L, salt solution – 1 mL/L [stock salt solution: CaCl_2_⋅2H_2_O – 150 mg, FeCl_3_ – 20 mg, MgSO_4_⋅7H_2_O – 2.46 g, distilled H_2_O – 100 mL], thiamine – 1200 mg/L, arginine‐HCl – 825 mg/L, biotin – 16.66 μg/L, B12 – 8.33 μg/L, serine – 1050 mg/L, lysine – 1250 mg/L, aspartic acid – 750 mg/L and distilled H_2_O) designed to reduce bacterial or fungal overgrowth that might be intracellular to the isolated cells and capable of emerging in traditionally used *Acanthamoeba* media (e.g. PYG or PG media) but simultaneously capable of maintaining cells as trophozoites (Mooney et al. 2024). Amoebae were maintained in 1 mL of MAD media at 25°C with heat‐killed *Escherichia coli* and monitored daily by microscope for approximately 1–2 weeks until reaching confluence. Cells were then dislodged using a cell scraper and via pipetting and transferred to a microcentrifuge tube to allow extraction of genomic DNA.

### 2.3 DNA Extraction

Extraction of genomic DNA was carried out using the DNeasy PowerSoil Pro Kits (Qiagen) as per the manufacturer's instructions. Quality and yield of DNA was assessed using the Nanodrop ND‐1000 and Qubit 3.0 fluorometer (Thermo Fisher Scientific) respectively and subsequently stored at −20°C until use in downstream applications.

### 2.4 Molecular Verification of *Acanthamoeba* Species

The presence of *Acanthamoeba* within samples was verified by PCR amplification of the genus‐specific segment ASA.S1 on the 18S rRNA gene using the primers JDP1 and JDP2 previously described (*T*_m_ 60°C; F – 5′‐GGCCCAGATCGTTTACCGTGAA‐3′, R – 5′‐TCTCACAAGCTGCTAGGGAGTCA‐3′), herein referred to as JDP PCR (Schroeder et al. 2001). JDP PCR was carried out using the Expand hi‐fidelity PCR system (MilliporeSigma) as per the manufacturer's instructions. Successful amplifications were ligated using the pGEM T‐Easy vector kit (Promega). Ligated products were transformed into competent DH5α *E. coli* , before selecting successful colonies using blue/white screening for sub‐culture, and finally plasmids extracted using the QIAprep Spin Miniprep Kit (Qiagen) as per the manufacturer's instructions. Sanger sequencing of the extracted plasmid insert was then carried out by Eurofins Genomics. Data were then screened using BLAST (NCBI) to verify and genotype the isolated amoebae. The evolutionary history was inferred by using the maximum likelihood method and Tamura–Nei model (Tamura and Nei 1993) and the tree with the highest log likelihood (−7899.02) selected (Felsenstein 1985). Initial tree(s) for the heuristic search were obtained automatically by applying neighbour‐joining and BioNJ algorithms to a matrix of pairwise distances estimated using the Tamura–Nei model, and then selecting the topology with superior log likelihood value. This analysis involved 37 nucleotide sequences, 3 generated as part of this study and 34 publicly available on the NCBI database. There were a total of 2169 positions in the final dataset. Evolutionary analyses were conducted in MEGA11 (Tamura, Stecher, and Kumar 2021).

### 2.5 Microbiome Analysis

Microbiome analysis was performed using the hypervariable target region V1–V3 (27F – 5′‐AGAGTTTGATCCTGGCTCAG‐3′ and 534R – 5′‐ATTACCGCGGCTGCTGG‐3′) (Johnson et al. 2019) of the 16S rRNA gene, commonly used in environmental community analyses (Baker, Smith, and Cowan 2003; Reller, Weinstein, and Petti 2007; Yu et al. 2008), to assess the shift in the prokaryotic population dynamics of the surrounding environment versus the recovered population after isolation of amoeba. Sufficient microbial DNA yield was not obtained from Laguna Verde and as such was excluded from downstream analysis. Successful samples were sequenced on an Illumina Platform and the raw data subjected to several quality control and filtration steps (FastQC, VSEARCH) before being sorted into operational taxonomic units (OTUs) using minimum entropy decomposition (Eren et al. 2013; Eren et al. 2015; Rognes et al. 2016). Assignment of the lowest taxonomic unit for OTUs was achieved using DC‐MEGABLAST. Alpha diversity was calculated using Chao1 and Faith's phylogenetic diversity metrics and beta diversity was calculated using the weighted and unweighted UniFrac distance.

## 3 Results

### 3.1 Isolation of Several *Acanthamoeba* Species at Distinct Geographic Locations

**FIGURE 3:**
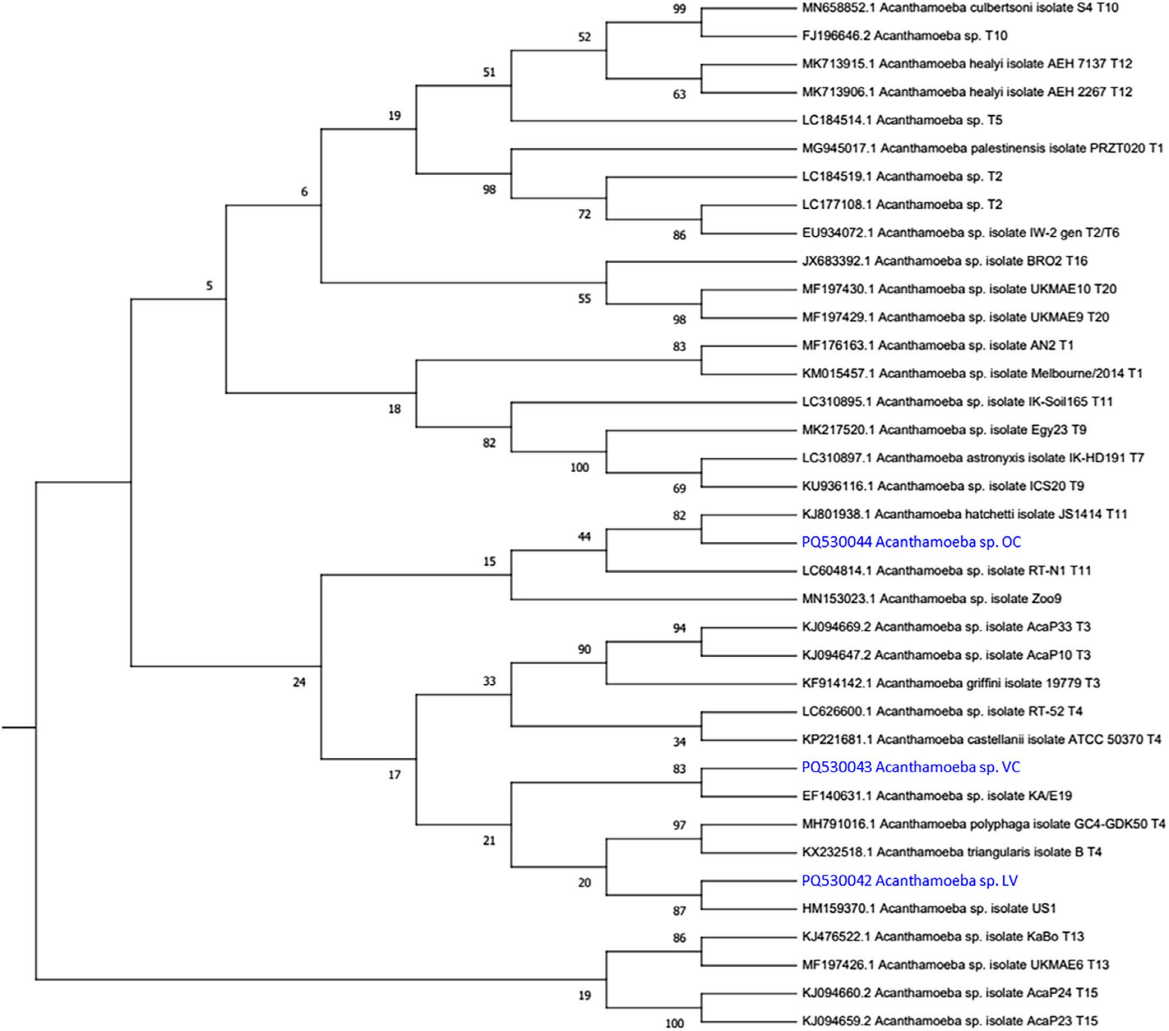
The evolutionary history for *Acanthamoeba* isolated as part of the present study (highlighted in blue) from Ojos de Campo (OC; PQ530044), Vega Colorada (VC; PQ530043) and Laguna Verde (LV; PQ530042), was inferred using the ASA.S1 region of the 18S rRNA gene relative to publicly available *Acanthamoeba* sequences encompassing the known genotypes (T1–T23). The branch lengths are proportional to the sequence divergence. Numbers along the branches are bootstrap values (1000 replicates).

Putative *Acanthamoeba* species were selected based on morphological characteristics and confirmation made using JDP PCR. Presence of *Acanthamoeba* at all sites was confirmed by culture and molecular screening, with viable trophozoites emerging from cysts at all sites, further evidencing the ubiquity of *Acanthamoeba* in the environment and its ability to survive under extreme conditions. Chemical conditions of these locations, although not measured as part of this study, are known to be high in lithium (Vignale et al. 2022) which is closely associated with elements such as potassium, magnesium, boron and sodium (Mauger et al. 2018; Mernagh et al. 2016), as well as occurring in salt lake brines within complex salts such as fluorides, chlorides and sulphates (Murodjon et al. 2020). These regions are also subject to higher ultraviolet radiation, desiccation, pH and arsenic than other saline systems (Farías 2020; McGenity and Oren 2012; Vignale et al. 2022). The genotyping of isolates of *Acanthamoeba* from each site was achieved using Sanger sequencing, by targeting the JDP region. The returned sequences were screened using BLAST for confirmation. Hits with the highest similarity were noted for each isolate and further confirmed using phylogenetic analysis. Sequence data from *Acanthamoeba* isolates originating from Vega Colorada (VC; PQ530043), Laguna Verde (LV; PQ530042) and Ojos de Campo (OC; PQ530044) were screened against known publicly available *Acanthamoeba* sequences encompassing a range of known genotypes (T1–T23) to discern the evolutionary closeness using maximum likelihood method (Figure 3). We found *Acanthamoeba* sp. isolates VC and LV clustered with *Acanthamoeba* of the T4 genotype, whilst the isolate OC clustered with *Acanthamoeba* of the T11 genotype (Figure 3, highlighted in blue).

### 3.2 Comparative Species Diversity of Overall and Intracellular Microbiomes

**FIGURE 4:**
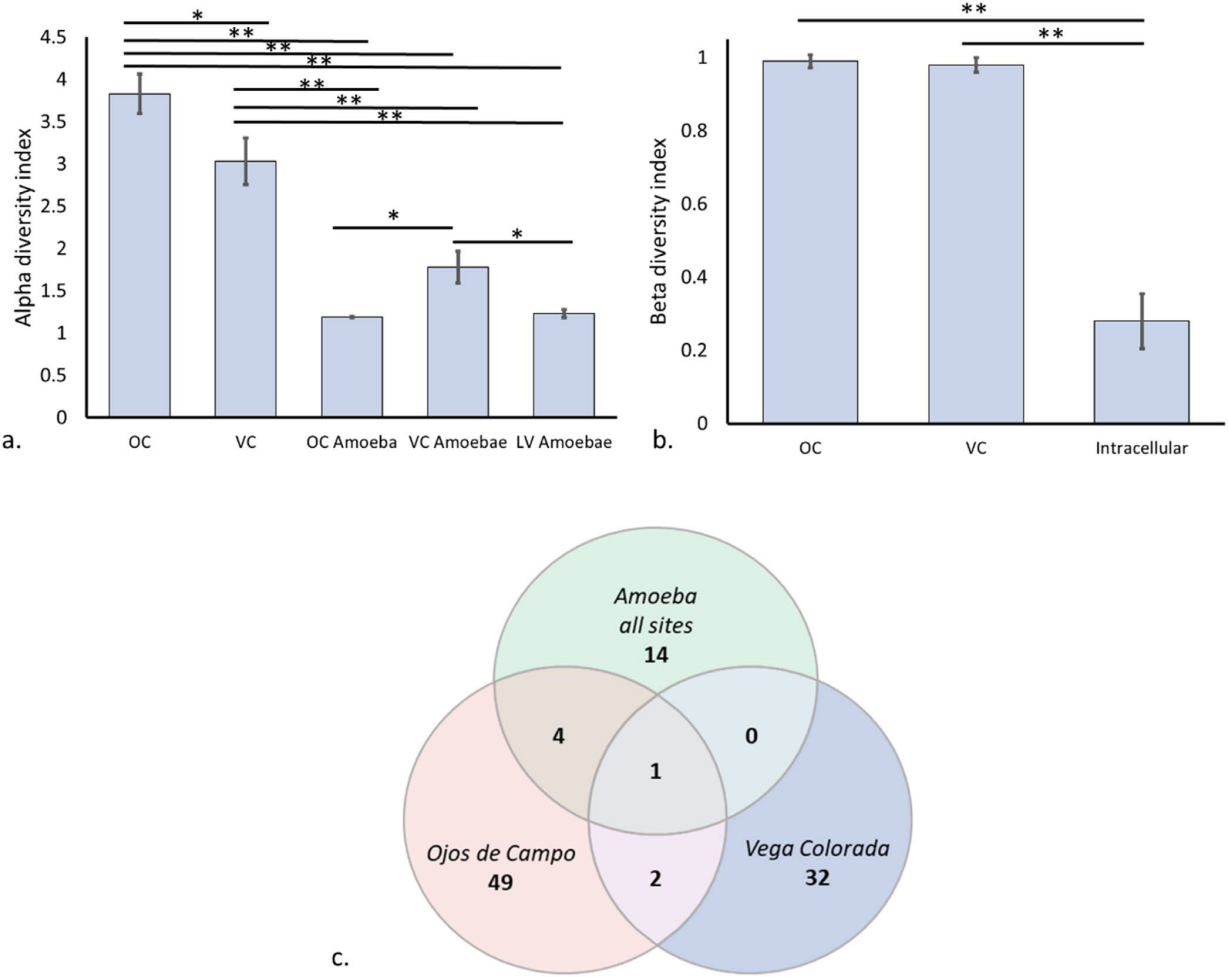
(a) Alpha diversity index of raw total samples and isolated amoeba samples. Raw total samples OC and VC were significantly more diverse than those isolated from amoebae. (b) Beta diversity of raw total samples and isolated amoeba samples. Raw total samples of OC and VC were much more diverse than those isolated from amoebae. (c) Shared OTUs from samples. Only one genus was shared amongst all samples, with the majority of genera being unique to sample site or amoebic isolation. Significance denoted by ‘*’ for *p* < 0.05 or ‘**’ for *p* < 0.01.

The prokaryotic microbiome residing inside of the isolated *Acanthamoeba* compared to the overall prokaryotic microbiome from two of the three sites: Vega Colorada and Ojos de Campo. Microbial DNA yield was insufficient from Laguna Verde to accurately describe the microbiome within this environment. Species richness at individual sites was determined by calculating the alpha diversity score for each sample (Figure 4a). As expected, microbial diversity was significantly higher in the total DNA extractions from the surrounding environments (Figure 4a: 3.83 and 3.03 for OC and VC respectively) than those from intracellular isolates (Figure 4a: 1.19, 1.78 and 1.23 for amoebae isolated at OC, VC and LC respectively, *p* < 0.01). The intracellular microbiome of OC and LV had the lowest alpha diversity scores, 1.19 and 1.23 respectively, showing no significant difference to each other (Figure 4a: *p* > 0.05), whilst amoebae isolated from VC showed slightly higher levels of diversity than the other amoebae (Figure 4a: 1.78, *p* < 0.05).

Beta diversity was calculated to determine differences in the diversity across all sites (Figure 4b). Unsurprisingly, total DNA from sample sites were highly diverse (Figure 4b: 0.99 and 0.98 for OC and VC respectively), with only 3 of the combined 84 genera detected at both locations (Figure 4c). Conversely, the diversity of bacteria from amoebic isolates was much lower (Figure 4b: 0.28), despite the environmental variability of the locations in which they were isolated (Vignale et al. 2022). Interestingly, 14 of the 19 taxa identified were entirely unique to the amoebic microbiome (Figure 4c), and 13 were conserved across all samples. Our results demonstrate the potential for amoebae to harbour an undetectable microbial community which can establish itself upon isolation and culture in new environments.

**FIGURE 5:**
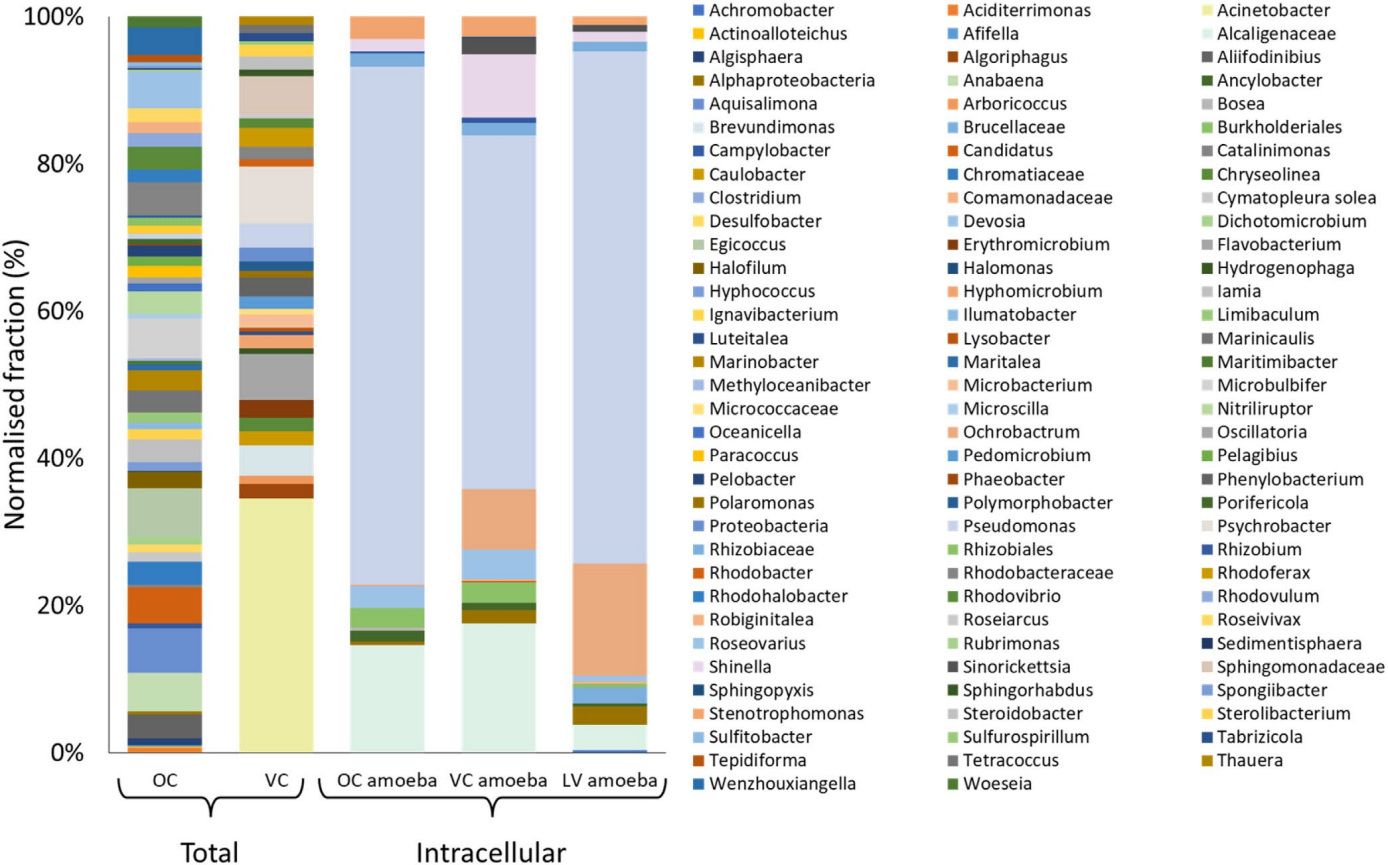
Molecular characterisation of extracellular (Total) and intracellular prokaryotes from three sites across the salt flat plains and lagoons of the Puna region of Catamarca province, Argentina. Total genomic DNA was isolated from Ojos de Campo (OC) and Vega Colorada (VC), and isolated amoebic cultures (Intracellular) from OC, VC and Laguna Verde (LV). Microbiome sequencing of the 16S rRNA gene region V1–V3 was undertaken to determine prokaryotic diversity. Sequence reads were assigned to the genus level where possible and abundance estimated using a normalised fraction of the OTU sequence reads.

Distribution for prokaryotes in each sample based on the sequencing of the 16S rRNA V1–V3 region is shown in Figure 5. A total of 102 unique OTUs were identified across all sites (Figure 4). Ojos de Campo was the most diverse of all samples and the most evenly distributed, with the most abundant genus, *Aquisalmonas*, comprising only 6% of all OTUs (Figure 5: OC). Vega Colorada also showed a relatively even distribution amongst most organisms with the exception of the genus *Acinetobacter*, which comprised 34.5% of all OTUs, followed by the genus *Psychrobacter* at 7.7% (Figure 5: VC). As shown previously (Figure 4), the intracellular microbiome was significantly less diverse than the total microbiome yet was almost entirely comprised of organisms not found during the screening of total DNA from the same environments. In keeping with previous studies on the intracellular microbiome of amoebae (Mooney et al. 2024; Rayamajhee et al. 2021), our analysis found a significant quantity of gram‐negative bacteria within the isolated amoebae, the most abundant of which were *Pseudomonas* spp., comprising 70.3%, 48.1% and 69.7% of all OTUs from amoebae isolated at sites OC, VC and LV respectively (Figure 5: Intracellular). The microbiome from *Acanthamoeba* isolated from sites VC and LV showed a higher composition of *Ochrobactrum* than those from site OC (Figure 5: 8.15% and 15.2% for VC and LV respectively vs. 0.2% for OC) whilst *Shinella* made up a larger proportion of the microbiome in VC amoebae than in those from LV or OC (Figure 5: 8.6% for VC vs. 1.7% and 1.4% for OC and LV respectively). OTUs corresponding to the family *Alcaligenaceae* were also shown to be much higher in VC and OC than LC (Figure 5: 14.6% and 17.6% for OC and VC respectively vs. 3.5% for LV). Associations between *Acanthamoeba* spp. and *Pseudomonas* spp. are well documented, however, the protective role and reduced detectability that amoebae seemingly provide to these bacteria is concerning given clinical significance of *Pseudomonas* (Botelho, Grosso, and Peixe 2019; Parcell et al. 2018; Reynolds and Kollef 2021).

## 4 Discussion

Despite an increased understanding of the importance of microbial community compositions in ecosystem health, relatively little is known surrounding the interactions that occur at this level between predatory protist species and the bacterial prey. Strategies that permit intracellular survival within organisms such as *Acanthamoeba* have emerged several times over evolutionary history and play an important role in shaping microbial communities (Flieger et al. 2018; Henriquez et al. 2021). In addition, this survival is clinically relevant, for example reducing detection, permitting pathogen transmission, and decreasing the efficacy of commonly used disinfectant strategies (Dobrowsky, Khan, and Khan 2017; García et al. 2007; Mooney et al. 2024; Okubo et al. 2018; Rayamajhee et al. 2022; Rayamajhee, Willcox, Henriquez et al. 2024; Sarink et al. 2020). Indeed, we have demonstrated here that *Acanthamoeba* spp. can harbour a ‘hidden’ prokaryotic microbiome. Isolation of amoebic cysts and culture in the absence of environmental stressors allows excystation and emergence of the protected bacteria that would elsewise go unnoticed. The survival of these organisms within protists poses an additional problem when considering the monitoring and mitigation of antimicrobial resistant organisms in the environment. Our research here demonstrates a potential for amoeba to act as a protective reservoir to a host of bacteria not previously detected using overall screening methods.

Overall, our microbiome analyses of the Vega Colorada and Ojos de Campo sites revealed distinct communities consistent with the environment they were identified from. The sub‐zero temperatures of Vega Colorada were unsurprisingly associated with a number of psychrophilic and physchrotolerant organisms. *Acinetobacter* dominated the community, perhaps due to their metabolic flexibility and tolerance for low temperatures (Radolfova‐Krizova, Maixnerova, and Nemec 2016; Yao et al. 2013; Zheng et al. 2018). In addition, various other psychrotolerant and psychrophilic organisms were identified, for example *Psychrobacter* spp., *Rhodoferax* spp. and *Polaromonas* spp., all of which are adapted to survival at low temperatures (Franzetti et al. 2013; Gawor et al. 2016; Kaden et al. 2014; Madigan et al. 2000; Welter et al. 2021). Furthermore, the dense vegetation of the ecosystem is supported by the presence of a number of bacteria capable organic matter decomposition and nutrient cycling within cold environments such as *Flavobacterium*, *Steroidobacter*, *Caulobacter*, *Hydrogenophaga* and *Thauera* (Berrios 2022; Jørgensen and Pauli 1995; Kolton et al. 2016; Ren et al. 2024; Wang et al. 2023; Xun et al. 2021). Conversely, Ojos de Campo is located in a salt flat, and microbes here are exposed to high levels of salinity, lithium and UV, and have limited oxygen and nutrient availability (Farías 2020; McGenity and Oren 2012; Vignale et al. 2022). The extreme conditions of this system are reflected in the microbiome, with a range of halophilic (e.g. *Halomonas*, *Rhodovibrio*, and *Aliifondinibius*) (Amiour et al. 2022; Oren 2024; Zhao et al. 2020) and anaerobic (e.g. *Clostridium*) (Dürre 2014) organisms dominating the community.

The amoebic isolations in this study were taken from harsh geochemical conditions on the Catamarca province, Argentina (Vignale et al. 2022). Successful isolation of distinct *Acanthamoeba* species from these environments demonstrates the ubiquity of the organism and their high tolerance for environmental extremes. In addition, the cysts isolated from these environments are capable of harbouring and protecting bacterial species that appear unable to survive extracellularly under these conditions but can re‐emerge when conditions are more favourable. Interestingly, our results show a significant variation in the detected organisms from the surrounding environment relative to the organisms detected upon isolation and conversion of amoebic cysts to the trophozoite stage, seemingly allowing a new microbiome to emerge which is unique to the overall screening. Diversity indices using the lowest OTUs between the sample site raw extractions and the amoebic isolations found that the alpha diversity of overall extractions was much higher than the amoebic isolates (> 3 relative to 1.19–1.78 for overall and amoebic isolates respectively), although 14 unique OTU assignments were noted from amoebae isolates. The beta diversity was estimated to be 0.99 and 0.98 for Ojos de Campo and Vega Colorada respectively. Interestingly, the beta diversity between amoebic isolates from all sites was much lower (0.28) suggesting that the populations within the species across these locations were consistent despite the external environment. *Acanthamoeba* cysts are a highly resistant and structurally sound double‐walled structure containing cellulose (Coulon et al. 2010; Johnston et al. 2009; Lemgruber et al. 2010), housing the trophozoite stage and likely a plethora of other microorganisms that reside within the trophozoite. It is likely that these cysts can limit the influence of the external environment on intracellular bacteria, which can emerge upon excystation into more hospitable environments should they arise.

Indeed, our observation that despite the variability in the surrounding environments they were isolated from, many of the taxa intracellular to the amoebae were common amongst all isolates. A total of 13 from the 19 amoebae‐associated bacteria were found in all isolates, more interestingly however is that 8 of those identified from the Argentine isolates were also identified in a similar study using *Acanthamoeba* isolated from an unnamed Scottish hospital (Mooney et al. 2024). In both studies, the dominant genus observed was *Pseudomonas*, well documented as having an intracellular association with *Acanthamoeba* (Dey et al. 2019; José Maschio, Corção, and Rott 2015; Leong et al. 2022; Mooney et al. 2024; Sarink et al. 2020; Spilker et al. 2004). *Pseudomonas* spp. have been recovered from *Acanthamoeba* in a range of studies, including multiple examples of intracellular survival during amoebic infection of the cornea (Hajialilo et al. 2019; Iovieno et al. 2010; Mohd 2017; Niyyati et al. 2015; Rayamajhee et al. 2021). This is concerning given the potential for several *Pseudomonas* species to cause infections in humans and lends credence to the idea that *Acanthamoeba* can act as a vector to potential pathogens in high‐risk areas (Henriquez et al. 2021). Furthermore, the presence of other taxa containing potential pathogens, for example Alcaligenaceae (e.g. *Achromobacter* spp., *Alcaligenes faecalis* ) (Huang 2020; Isler et al. 2020) and Brucellaceae (e.g. *Brucella* spp.) (Głowacka et al. 2018) from amoebic isolates but not from the total environment is highly concerning and further emphasises the need for considering interkingdom relationships within current monitoring approaches. In keeping with our analysis, and in addition to *Pseudomonas*, several other bacteria detected in this study have also been identified in various amoebic cultures globally using culture or molecular approaches, including *Candidatus* spp., *Stenotrophomonas maltophilia* , *Achromobacter* spp. and *Sinorickettsia* spp., as well as various Burkholderiales and Rhizobiales species (Choi et al. 2024; Rayamajhee et al. 2021; Rayamajhee, Willcox, Sharma et al. 2024). Despite the shared taxa, it should be noted that the amoebic isolation procedure has the potential to influence the dynamics of the bacterial microbiome, and more research is required to develop a more comprehensive approach to detecting hidden microbiomes.

Given the range of organisms identified within this study, it is interesting to consider the variety of phagocyte resistance strategies that are likely utilised. Whilst little is known surrounding the mechanisms of intracellular survival in *Acanthamoeba*, the similarities between these organisms and human immune cells makes for a useful comparison (Rayamajhee et al. 2022; Siddiqui and Khan 2012). For example, in *Pseudomonas* spp., intracellular survival is partially achieved through inhibition of reactive oxygen species used to breakdown organic materials within the phagosome (Vareechon et al. 2017), whilst *Brucella* spp. can functionally modify the phagosome to permit intracellular replication (Gorvel and Moreno 2002). It is now increasingly considered that the predator–prey relationship between amoebae and bacteria has selected for many organisms resistant to phagocytosis, and as such this selection has inadvertently selected for bacteria capable of evading human immune responses (Henriquez et al. 2021; Rayamajhee et al. 2022). It is not unsurprising then that many of the taxa present within amoebae are also capable of causing infection in humans and animals, and perhaps by furthering our understanding of these interactions in the environment we might be better equipped to combat these bacteria clinically, or better prepared for the emergence of novel pathogens. The potential for many of these bacteria to survive within amoebae for prolonged periods, and the apparent universality of many species, suggests that the amoebic microbiome is not a reflection of the surrounding environmental microbiome, but instead it is a distinct, and highly mobile, ecological niche.

Further research into the risk factors of intracellular survival is required, however, we have shown here that certain protist species have the potential to limit exposure of bacteria to external pressures and can act as a reservoir for these organisms, permitting recolonization upon removal of the associated stressors. This is especially relevant in clinical settings given the reduced detectability of potential pathogens and the capabilities of amoebae to survive higher levels of antimicrobial treatments. Briefly, the role of predatory protists in harbouring bacteria should be considered when implementing detection and mitigation strategies.

## Author Contributions

**Ronnie Mooney:** methodology, conceptualization, data curation, formal analysis, investigation, writing – review and editing, writing – original draft. **Kiri Rodgers:** methodology, conceptualization, data curation, writing – review and editing, writing – original draft. **Sandro Carnicelli:** investigation, funding acquisition, project administration, resources, writing – review and editing. **Matías E. Carnevale:** investigation, project administration, resources, writing – review and editing. **Maria Eugenia Farias:** conceptualization, investigation, project administration, resources, writing – review and editing. **Fiona L. Henriquez:** conceptualization, methodology, data curation, investigation, supervision, resources, project administration, visualization, funding acquisition, writing – review and editing, writing – original draft.

## Conflicts of Interest

The authors declare no conflicts of interest.

## Data Availability

The data that support the findings of this study are available from the corresponding author upon reasonable request.
